# Pennsylvania Hospital

**Published:** 1846-05

**Authors:** 


### THE WESTERN JOURNAL.

NEW SERIES.—VOL. V.—NO. V.

LOUISVILLE, MAY 1, 1846.

### PENNSYLVANIA HOSPITAL.

Our correspondent from Philadelphia writing of the Pennsylvania Hospital gives the following details which may be of interest to our readers.

Besides the regular inmates of the wards, a number of indigent persons daily present themselves for the purpose of having their diseases investigated and prescribed for, which at this season is done in the hall of the first building; during the sessions of the medical schools, if any interesting cases are presented they are brought before the students in a room appropriated to that purpose. Among those on Saturday, I saw a case of Carcinoma, originally affecting the lower lip near the right angle of the mouth, but extending now along the inner margin of the right side of the lower jaw, exposing in its ravages over this surface the sublingual salivary glands, and causing a constant flow of saliva through the opening. Commencing at the lower part of the ear, on the same side, and extending the whole length of the parotid gland, exposing also this body, is another ulcer. The patient is a native of Ireland, 55 years of age; two years ago he observed a small wart upon his lower lip, which, together with a portion of the lip, was removed by some surgeon near Dublin; soon afterwards he sailed for this country and had hardly reached here before the disease reappeared a little to the right of its original seat. Of course, nothing was done for the miserable man, and as he walked out of the hospital he looked the picture of despair.

In the wards were a great number of fractures, particularly of the lower extremities. Many of these cases were in the persons of seamen, who met with the accidents some weeks ago while at sea during those severe gales of which you have seen an account. Most of these were difficult and aggravated cases, some of them of long standing, from having, as I said, occurred at sea where the men had not the advantage of surgical attendance.

I saw also several cases of opacity of the cornea, two or three of which supervened upon attacks of small-pox. The treatment, by leeches, nitrate of silver, and sulphate of copper, was affording decided benefit. I cannot forbear to speak of the kindness of manner and sympathy manifested by Dr. Norris, at present the attending physician, towards all the patients.

In the afternoon I visited the Anatomical Museum belonging to the University. This museum, being supported by annual appropriations made by the trustees, presents an air of great cleanliness and neatness. The alcohol contained in the thousand jars is removed every summer if at all discolored, fresh liquor supplied, the jars again covered with their six layers of cloth, varnished, labelled, and set away in their places. All the dried preparations are attended to with equal care, and by timely labor the ravages of the worms are prevented. The untiring industry and great skill of Professor Horner are seen in the large number, rareness and beauty of the specimens, preparations, and dissections. In one of the cases are to be seen casts in plaster of Paris of two limbs of persons who had anchylosed knee joints, taken both before and after the operation for that deformity, which consists in making the V excision, first proposed, I believe, by Dr. Barton. The casts taken before the operation show limbs distorted and wholly useless—those after, limbs improved in symmetry, and not less so in usefulness.

I have thought that a brief notice of the operation not long since performed by Dr. J. K. Rodgers for aneurism of the subclavian artery, of which you have seen notices in the New York journals, might not be uninteresting to you.

The subject of this operation, a man aged 35 years, was admitted into the New York Hospital in December, 1845, with aneurism of the subclavian artery of the left side. Dr. Rodgers applied a ligature just inside of the scalenus anticus muscle; pulsation ceased immediately, and the ligature came away on the thirteenth day, when a slight hemorrhage began, and persisted until the patient died on the fifteenth day after the operation.

The following are the appearances presented by the post-mortem examination: the tumor was found perfectly consolidated, as was also the artery from the ligature to the aorta; there was more than an inch of firm coagulum between the ligature and the heart; the axillary artery leading from the tumor was filled with a finely organized fibrinous clot, which adhered closely to the parietes of the vessel. The hemorrhage which caused the fatal termination appears to have proceeded from the vertebral artery, which was just on the distal side of the ligature.

The fine coagulum formed in the subclavian on its proximal side, of course, would have prevented secondary hemorrhage.

This case to my mind clearly demonstrates the feasibility of the operation in question, for had the vertebral artery been tied along with the subclavian, which Dr. Rodgers would have done had not that artery been unusually deep-seated, the patient would almost without doubt have recovered.

The specimen exhibiting all the appearances that I have just described is in the hospital museum where, after having had a history of the case from my friend, Dr. Sayre, I noted down nearly all that I have written. It is one of those cases in which the surgical world at present feels an especial interest; and certainly, I think, authorizes the hope that by the operation proposed—the application of a ligature to the vertebral and subclavian arteries at the same time—the left subclavian may in many instances be successfully ligatured.

D. W. Y.

March 29th, 1846.

